# Effects of icariin on long noncoding RNA and mRNA expression profile in the aortas of apoE-deficient mice

**DOI:** 10.1042/BSR20190855

**Published:** 2019-07-26

**Authors:** Yibing Zhang, Rui Xu, Xiangjun Li, Qi Tan, Peng Huang, Yang Zhang, Meng Qin, Liqun Ren

**Affiliations:** 1 Department of Pharmacology and Toxicology, Jilin University School of Pharmaceutical Sciences, 1163 Xinmin Ave, Changchun 130021, China; 2 Department of Ophthalmology, The First Hospital of Jilin University, Changchun 130021, Jilin, China; 3 Norman Bethune College of Medicine, Jilin University, 828 Xinmin Ave, Changchun 130021, Jilin, China; 4 Department of Pathology, Third Hospital of Jinzhou Medical University, Jinzhou 121000, China

**Keywords:** atherosclerosis, bioinformatics analysis, icariin, long non-coding RNA, microarray

## Abstract

***Objective***: The beneficial effects of icariin (ICA) in ameliorating atherosclerosis (AS) are well known, but the underlying protective mechanism has not been fully elucidated. The present study aimed to investigate altered long noncosing RNA (lncRNA) and mRNA expression profiles in ApoE^−/−^ mice after ICA treatment.

***Method***: The atherosclerotic plaque area was evaluated on high-fat diet (HFD)-induced ApoE^−/−^ mice treated with either ICA or vehicle. LncRNA and mRNA integrated microarrays was performed on aortic tissues. Gene Ontology (GO) and Kyoto Encyclopedia of Genes and Genomes (KEGG) pathway analysis were utilized to explore the significant function and pathway of the differentially expressed (DE) mRNAs, global signal transduction network were constructed to select key mRNAs, and lncRNA–mRNA co-expression network was built to find out the interactions between lncRNA and mRNA. Quantitative real-time PCR (qPCR) was used to further validate the expressions of selected lncRNAs and mRNAs.

***Results***: Administration of ICA significantly reduced plaque size after 12 weeks (*P*<0.05). A total of 1512 DE lncRNAs and 2059 DE mRNAs were identified. The mRNAs: protein kinase C, β (Prkcb), Cyp2c65, Mapk10, Calmodulin 5 (Calm5), Calmodulin-like 3 (Calml3) and Camk4 were selected as hub mRNAs, the correlated lncRNAs in co-expression network were identified as important regulatory lncRNAs. The identified target pairs such as lncRNA-NONMMUT000659/Prkcb may play critical roles in AS development mediated by ICA.

***Conclusion***: Taken together, our study highlights a panel of DE lncRNAs and mRNAs that could explain the molecular mechanism of ICA’s anti-atherosclerotic effects. The work lays a foundation for subsequent genes functional researches, which could contribute to provide new therapeutic targets for AS.

## Introduction

Atherosclerosis (AS), characterized by progressing atherosclerotic plaques formation and luminal narrowing of arteries, underpins coronary artery disease (CAD) with high mortality worldwide [1]. Despite great advances in therapeutic agents such as statin, CAD remains a healthcare and economic burden. Moreover, potential adverse effects of statin application make several patients stop receiving statin therapy [2,3]. Hence, identifying novel curative strategies as well as new biomarkers for therapeutic targeting in AS are desired.

Medicinal herbs emerge as alternative and complementary options with high efficiency and less side effects for AS management [4]. Herba Epimedii has been documented in the Chinese Pharmacopoeia (2015) as a traditional Chinese herb has extensive clinical indications [5]. Icariin (ICA) (C_33_H_40_O_15_; molecular weight: 676.67 g/mol), the main active ingredient of Herba Epimedii, has been previously confirmed to have a wide range of pharmacological and biological activities, including antioxidant effects, immunoregulation, anti-osteoporotic activity, and improving cardiovascular function [6–8]. Pieces of evidence have shown that ICA appears to be beneficial for AS *in vivo* and *in vitro* models [9–11]. Our recent study demonstrated that ICA was a concentration-dependent agent in protecting human vascular endothelial cells (HUVECs) against ox-LDL-induced injury [12]. However, the detailed molecular mechanisms of its therapeutic capacities remain unclear.

Noncoding RNAs (ncRNAs), a group of RNA molecules mainly containing long ncRNAs (lncRNAs) and microRNAs (miRNAs) are emerging as powerful regulators of cardiovascular disease [13,14]. Presently, cumulative studies have concentrated on the underlying RNA-mediated gene regulation of medicines [15,16]. To date, some miRNAs have been characterized in gene modulations of ICA [17,18]. However, the expression pattern and function of lncRNAs in beneficial effects of ICA are still unclear. LncRNAs, the rising stars in biology, are capable of affecting pathogenic processes of AS by mediating cellular apoptosis and proliferation, lipid metabolism, and inflammations [19,20]. Thus, whether ICA has effects on the expression of lncRNAs and targeted genes is reasonable. The biological functions and molecular mechanisms of lncRNAs in AS remain to be determined, particularly in relation to the anti-atherosclerotic effect of ICA.

In this context, the present study was designed to assess the effect of ICA on AS development in ApoE^−/−^ mice. Moreover, we investigated the lncRNA and mRNA expression differences in the aorta of ApoE^−/−^ mice with or without ICA treatment. By lncRNA and mRNA integrated microarrays, we conducted comprehensive bioinformatics analysis to explore the gene functions and interactions. On ground of this, we performed quantitative real-time PCR (qPCR) to further validate the microarray results.

## Materials and methods

### Animal treatment

All experiments on mice were approved by Animal Care and Use Committee of Jilin University (Permit Number: 2016-0304) and in accordance with the National Research Council’s Guide for the Care and Use of Laboratory Animals. ApoE^−/−^ mice on C57BL/6J background (male, 8 weeks old), purchased from Beijing Huafukang Bioscience Co., Inc., Institute of Laboratory Animal Science, Chinese Academy of Medical Sciences (Beijing, China, certificate no. SCXK (Jing) 2014-0004), were housed in Institute of Regeneration Medical Science, School of Pharmaceutical Sciences, Jilin University. All mice were maintained in groups under a 12-h/12-h light–dark cycle in temperature controlled environment (22 ± 2°C) with free access to food and water. After adapting in lab for a week, a total of 24 ApoE^−/−^ mice were randomly divided into an ICA-treated group and an AS model group (*n*=12 for each group). Accelerated AS was induced by feeding the mice a high-fat diet (HFD), which comprised 20% fat and 1.25% cholesterol (D12108C, Research Diets Inc). Every morning at 9:00, each group received 0.2 ml ICA or carboxymethylcellulose sodium (CMC-Na) by gavage, for 12 successive weeks. ICA was purchased from Vic’s Biological Technology Co., Ltd (Sichuan, China). According to the ratio of body surface between mice and rats, the 40 mg/kg/day ICA was chosen as intervening dosage as described in our previous study [21].

### Determination of serum lipid concentration

Following 12 weeks of HFD feeding, mice were killed and blood was collected by cardiac puncture. Serum was prepared from each blood sample by centrifugation at 3000 rpm for 15 min. Serum levels of total cholesterol (TC), triglycerides (TG), high-density lipoprotein cholesterol (HDL-C), and low-density lipoprotein cholesterol (LDL-C) were determined using an auto-analyzer (Hitachi 917,Tokyo, Japan) according to the manufacturer’s instructions.

### AS lesion assessment

After collection of blood samples, six mice were randomly selected from each group for *en face* aorta Oil Red O staining. As the published protocol described [22], after perfusion with phosphate-buffered saline (PBS) followed by 4% paraformaldehyde via the left ventricle, the heart and aorta were removed, and then whole aortas were rapidly dissected from the aortic root to the iliac bifurcation with adventitial fat and connective tissue totally removed, opened the full-length aortas longitudinally and stained with Oil Red O for *en face* morphometric analysis of the atherosclerotic lesions. Images were captured with Canon EOS760 IS digital camera (Tokyo, Japan). The lesion size of the aorta was analyzed by Image-Pro Plus 6.0 software (NIH Image, U.S.A.). Data were presented as mean percent of lesion area of the total aorta area ± SD. All investigators handling the record collection and analysis were blinded to group.

### LncRNA/mRNA microarray

For Affymetrix microarray profiling, the total RNA was isolated from whole aortic tissue sample per ApoE^−/−^ mouse by TRIzol reagent (Invitrogen, Carlsbad, USA), digested by DNase treatment, and purified with an RNeasy Mini Kit (Qiagen, Hilden, Germany), per manufacturers protocol. The amount and quality of RNA were determined by a UV-Vis Spectrophotometer (Thermo, NanoDrop 5000, U.S.A.) at 260 nm absorbance. The mRNA expression profiling was measured by Clariom™ D solutions for Mouse (Affymetrix GeneChip, Santa Clara, CA), containing 66100 gene-level probe sets. The microarray analysis was performed by Affymetrix Expression Console Software (version 1.2.1). Raw data (CEL files) were normalized at the transcript level using robust multi-array average method (RMA workflow). Median summarization of transcript expressions was calculated. Gene-level data were then filtered to include only those probe sets present in the ‘core’ metaprobe list representing RefSeq genes.

### Bioinformatics analysis

#### Gene Ontology and Kyoto Encyclopedia of Genes and Genomes analyses

The functions of most lncRNAs are unknown and may be learned from their associated protein-coding genes. Thus, we performed Gene Ontology (GO) and Kyoto Encyclopedia of Genes and Genomes (KEGG) pathway analyses on differentially expressed (DE) mRNAs between treatment and model groups. GO analysis was applied to analyze the major function of the specific genes with significant differences in the representative profiles of DE genes. GO analysis can uncover the gene regulatory network on the basis of biological processes and molecular function and organize genes into hierarchical categories [23]. Specifically, the chi-square test and two-sided Fisher’s exact test could classify the GO category. Enrichment provides a measure of the significance of the function: as the enrichment increases, the corresponding function is more specific, which helps us to find those GOs with more concrete function description in the experiment. Pathway analysis could locate the significant pathways of the DE genes according to KEGG. Significant pathways were selected by Fisher’s exact test and chi-square test [24] and the standard of difference was *P*<0.05.

#### Global signal transduction network

Global signal transduction network was constructed to demonstrate the interaction between DE genes. KEGG database was used to analyze the functional gene interactions in the pathway. Networks were presented as graphs using Cytoscape software, each gene corresponded to a node, the nodes connected by an edge. The degree was defined as the number of links from one node to others, and genes with higher degrees occupied more important positions in the signaling network. Likewise the character of a gene was also described by betweenness centrality, which could assess a gene’s centrality in the network. Gene interactions could be drawn based on the statistics. Thus, the signal transduction network analysis was a method to select the core genes which had powerful capacity to modulate adjacent genes [25,26].

#### LncRNA–mRNA co-expression network

According to the normalized signal intensity of specific expression in mRNAs and lncRNAs, lncRNA–mRNA co-expression network was built to identify the correlations between lncRNA and mRNA. Cytoscape software was used to draw the co-expression networks. For each pair of genes, we calculated the Pearson correlation coefficient (PCC) and selected significant correlation pairs to construct the network. Only the strongest correlated (PCC ≥ 0.99) genes were presented in the co-expression network in which the specific lncRNA and mRNA were connected by an edge indicating either positive or negative correlation [27,28].

### qPCR

qPCR was performed to validate some of the core DE mRNA and the related lncRNAs. Total RNA was extracted using TRIzol reagent (Invitrogen) following purification with an RNeasy kit (Qiagen). The cDNA was synthesized by reverse transcription using a PrimeScript™ RT reagent kit with random primers according to the manufacturer’s protocols (Takara, RR036A). Then, qPCR was carried out using SYBR Premix ExTaq™ II (Tli RNaseH Plus, RR820). The 2^(−ΔΔ*C*^_T_^)^ method was used to quantify the relative expression of each gene, using GAPDH as an internal control [29]. All the experiments were conducted in triplicate and were repeated three times. All qPCR primer sequences are shown in Table 1.

**Table 1:** Primers used for qPCR

LncRNA or mRNA	Forward primer (5′–3′)	Reverse primer (5′–3′)
Gm8080	TGAATTTGCCTCGTCTTG	CTCCAGGTAAGTTGTGCC
NONMMUT051065	ACAGTCAGGAAGGCAGGT	AAGCAGATGGTCAGGGTC
Gm5327	CAGGCAGAACAACAGATACA	TCCCAGAGCTGAAAGTGA
NONMMUT005483	GCATCTCCTCTTGTGCCTCTTC	CTCCCAGCCTTGCTTAGTCG
Gm2904	TTAAACCAGACAGCCAGTA	AACAGATTTCCAGCAACA
NONMMUT031625	AGAATTACTGGGAGTTGC	TGTTGGGATTTGGTTTAT
NONMMUT031859	CACCAATCCCTGACACTA	CCTGGAGCTACGTCTAAT
NONMMUT000659	AAAGGCCCAGGCTTTCCT	CAGCACCGCCGTATCACA
Ighv8-14	CAGTAGTATGTGGCAGTATCT	CTACAACCCATTCCTGAG
Cyp2c65	CTTTGGCTGTGACTTCTG	GGGACTTTATTGATTGTTTC
Calm5	GCTCCTGCTCTTTAGTGT	ATGTCTCACGGGTTTACT
Calml3	GGTGCCGTTTCCATCTTT	CACTGTCATGCGGTCCCT
Prkcb	CGGTAAATAATGCCCTTG	CCGCCTGTACTTTGTGAT
Mapk10	GACTACAATGTTACTGGGTTTT	ACGAGCGGATGTCTTACT
Camk4	TGTTGGAGGGCTTGAAAT	TCTATGACGAGCGAGGTG

Abbreviation:Prkcb, protein kinase C, β.

### Statistical analysis

Statistics in microarray and bioinformatics analyses were performed by one-way analysis of variance (ANOVA) using Affymetrix® Expression Console™ TAC (Affymetrix® Expression Console™), followed by the least significant difference (LSD) test; statistics of body mass, serum lipid levels, atherosclerotic lesion area, and gene expression were performed by Student’s *t* test, using SPSS 16.0 software (SPSS, Chicago, IL, U.S.A.). *P*<0.05 was considered as statistically significant.

## Results

### Body mass and serum lipid concentrations

After 12 weeks, there was no difference in body weight between the ICA-treated and model mice (*P*>0.05). Compared with the AS model group, TC and LDL-C levels were significantly lower in serum of the ICA group (*P*<0.05). TG and HDL-C levels did not differ among groups (Table 2).

**Table 2:** Effects of ICA on weight and lipid profiles

	Weight (g)	TC (mmol/l)	TG (mmol/l)	HDL-C (mmol/l)	LDL-C (mmol/l)
Model	31.61 ± 1.82	29.06 ± 3.72	4.37 ± 0.72	3.22 ± 0.96	12.68 ± 2.42
ICA	30.39 ± 1.45	22.97 ± 2.83*	4.16 ± 0.35	2.61 ± 0.67	9.25 ± 1.72*

Data are shown as means ± SD. *P<0.05 vs. Model.

### ICA reduced AS lesion area in ApoE^−/−^ mice

**Figure 1:**
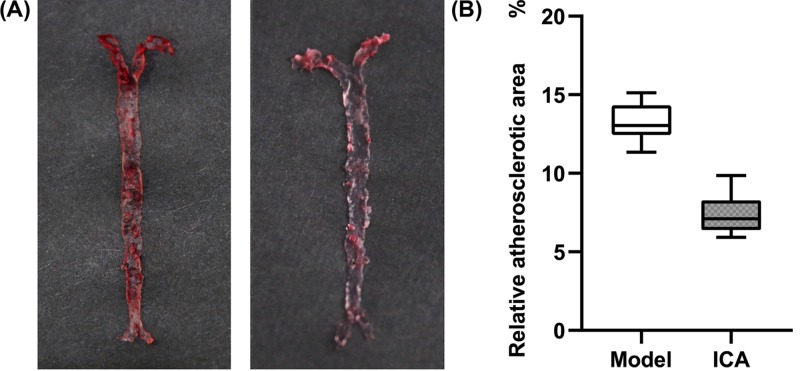
ICA attenuates atherosclerotic plaque formation in HFD-fed ApoE^−/−^ mice (**A**) Representative photographs of *en face* Oil Red O staining in aorta. (**B**) Box plot of statistics for atherosclerotic plaque area for all samples (Model: *n*=6; ICA: *n*=6). Ratio of plaque area to the whole aorta area in ICA (40 mg/kg/day) groups were significantly reduced compared with model group, *P*<0.05. Results are displayed as the mean ± SD.

To confirm whether ICA could suppress the progression of AS *in vivo*, we examined the effect of ICA on atherosclerotic lesion formation in aortas. As we established the atherosclerotic model in ApoE^−/−^ mice with the prolonged dietary routine of HFD, the AS model group developed obvious advanced atherosclerotic lesions with Oil Red O in the aortic tree. Remarkably, the ratio of the total area of atherosclerotic lesions, relative to that the luminal surface area of the entire aorta was significantly reduced by 5.85% in the ICA-treated of ApoE^−/−^ mice, compared with the model group of mice (*P*<0.05, Figure 1).

### DE lncRNAs and mRNAs in ApoE^−/−^ mice

**Figure 2:**
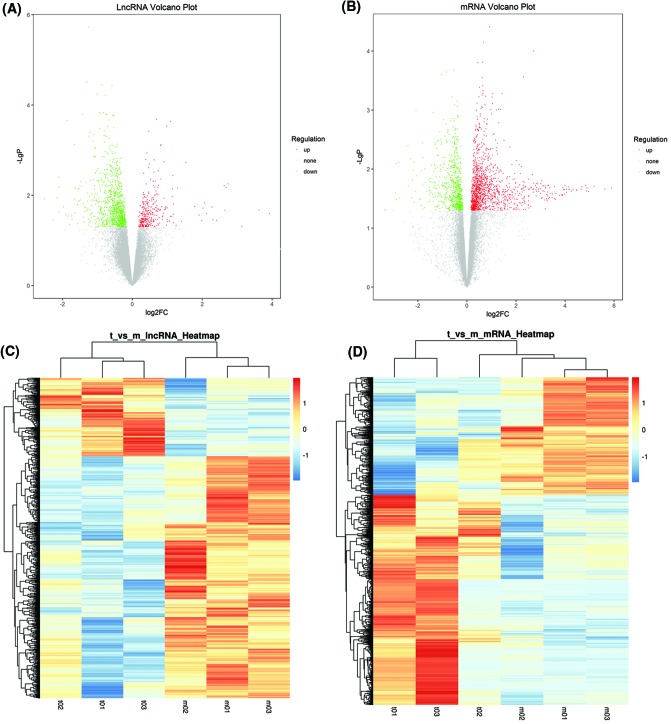
Expression of DE lncRNAs and mRNAs in ICA-treated and model groups Values plotted are mean normalized signal values (log2 scaled) for control (x-axis) and experimental (y-axis) groups. Red and green points correspond to 2.0-FCs up/down, respectively, and indicate *P*<0.05. (**A**) Volcano plots of DE lncRNAs. (**B**) Volcano plots of DE mRNAs. DE lncRNAs hierarchically clustered, each column represents a tissue sample and every row represents an lncRNA/mRNA probe. Red indicates high relative expression and blue indicates low relative expression. (**C**) Hierarchical clustering of DE lncRNAs. (**D**) Hierarchical clustering of DE mRNAs.

DE mRNAs and DE lncRNAs were then identified through fold change (FC) as well as *P*-value calculated with *t* test. The threshold set for up- or down-regulated RNAs was a FC >1.1 and a *P*-value <0.05. In total, 1512 lncRNAs displayed differential expression between ICA-treated and model groups, including 330 up-regulated and 1182 down-regulated lncRNAs. Of 2059 mRNAs that showed differential expression, 1317 were up-regulated and 742 were down-regulated. Volcano plot filtering was used for assessing gene expression variation between ICA-treated and model groups. Next, hierarchical clustering was applied to present the diacritical lncRNA and mRNA expression patterns among the samples. The data suggested that the expression of lncRNAs and mRNAs in ICA groups significantly differed from those in model controls (Figure 2).

### GO and KEGG analyses (functional annotation of key DE mRNAs)

**Figure 3:**
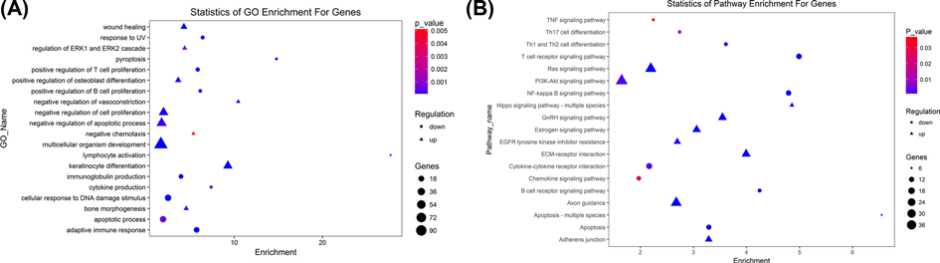
GO and KEGG analyses of DE mRNAs A total of 2059 DE mRNAs are chosen in GO and KEGG pathway analysis. The x-axis shows the enrichment of these mRNAs. The y-axis shows GO category or pathway. The size of the node’s area represents the number of genes, and the color of node represents *P-*value. Triangles indicate up-regulation and circles indicate down-regulation. (**A**) Significant changes in gene function by GO analysis. (**B**) Significant pathways induced by ICA.

GO enrichment and KEGG pathway analyses were performed to analyze the significant gene function. On the biological processes level, GO analysis (Figure 3) revealed that key DE mRNAs were enriched in up-regulated GO functions, including keratinocyte differentiation, multicellular organism development, wound healing, negative regulation of cell proliferation, positive regulation of osteoblast differentiation, negative regulation of vasoconstriction, negative regulation of apoptotic process, bone morphogenesis, regulation of extracellular signal-regulated kinase (ERK) 1 (ERK1) and ERK2 cascade, and negative chemotaxis. Down-regulated GO functions included adaptive immune response, cellular response to DNA damage stimulus, positive regulation of T-cell proliferation, immunoglobulin production, response to UV, lymphocyte activation, pyroptosis, positive regulation of B-cell proliferation, cytokine production, and apoptotic process. KEGG analysis showed that key DE mRNAs were enriched by up-regulated pathways, including ECM–receptor interaction, GnRH signaling pathway, Axon guidance, Focal adhesion, Estrogen signaling pathway, Ras signaling pathway, Adherens junction, EGFR tyrosine kinase inhibitor resistance, Hippo signaling pathway—multiple species, and PI3K-Akt signaling pathway. Down-regulated pathways included T-cell receptor signaling pathway, NF-κB signaling pathway, Apoptosis, B-cell receptor signaling pathway, Apoptosis—multiple species, Th1 and Th2 cell differentiation, Cytokine–cytokine receptor interaction, Th17 cell differentiation, Chemokine signaling pathway, and TNF signaling pathway.

### Global signal transduction network

**Figure 4:**
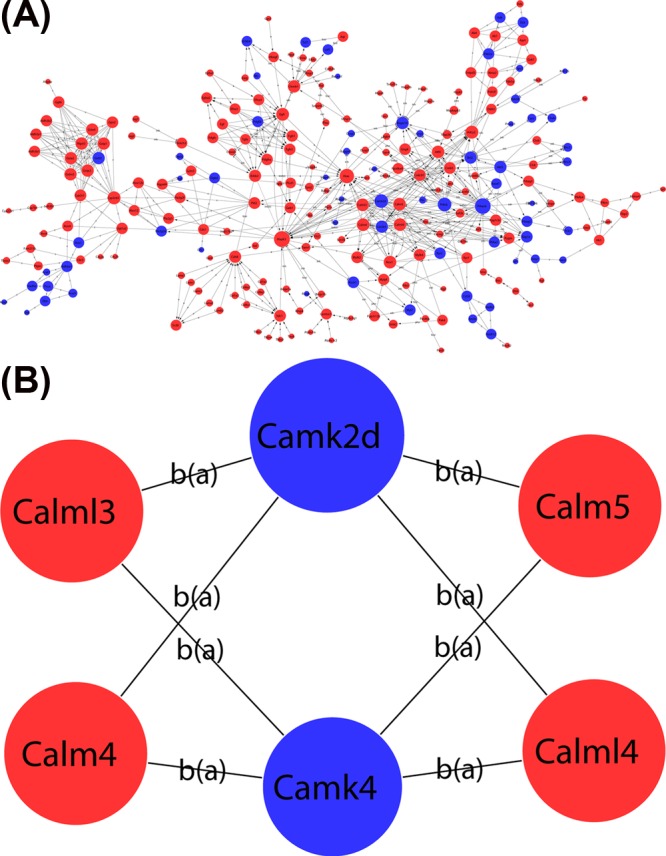
Global signal transduction network network analysis of DE mRNAs In the network, nodes represent mRNAs, the size of the node’s area represents the value of degree, red color indicates up-regulation and blue color indicates down-regulation. The nodes connect by an edge. The indicators a, b, c, p, inh, ind(e) are abbreviations of activation, binding, compound, phosphorylation, inhibition, indirect effect, respectively. (**A**) The whole global signal transduction network of DE mRNAs. (**B**) Magnified (A) portion; subnetwork of key mRNAs.

Global signal transduction network was performed to identify the core mRNAs. As shown in Figure 4, there was a mount of 346 key genes which were obtained in the signal-net. According to the network, the top 15 genes ranked by degree were Prkacb (protein kinase, cAMP dependent, catalytic, β), Mapk3 (mitogen-activated protein kinase (MAPK) 3, also known as Erk1), Hras (Harvey rat sarcoma virus oncogene), Camk2d (calcium/calmodulin-dependent protein kinase II, δ), Prkcb (protein kinase C, β), Cyp2c65 (cytochrome P450, family 2, subfamily c, polypeptide 65), adenylate cyclase 2 (Adcy2), epidermal growth factor receptor (Egfr), Mapk10 (also known as JNK3), Calm4 (calmodulin 4), Calm5 (calmodulin 5), Calml3 (Calm-like 3), Calml4 (Calm-like 4), Ctnnb1 (cadherin-associated protein, β 1), and Camk4 (calcium/calmodulin-dependent protein kinase IV). Meanwhile as shown in Table 3, Prkcb, Prkacb, Mapk3, Hras, Adcy2 and Egfr, which connected the most genes involved in the network were among the top ten genes ranked by betweenness centrality.

**Table 3:** The top ten mRNAs ranked by betweenness centrality after analysis of global signal transduction network

Gene symbol	Betweenness centrality	Degree	Style
*Pld2*	0.041852039	9	Up
*Plcd1*	0.041843613	4	Up
*Dgkd*	0.041746714	5	Down
*Agpat4*	0.041472868	5	Up
*Prkcb*	0.039888777	18	Down
*Prkacb*	0.030781113	31	Down
*Mapk3*	0.027558157	29	Up
*Hras*	0.025658083	22	Up
*Adcy2*	0.017082838	16	Up
*Egfr*	0.009876068	15	Up

### LncRNA–mRNA co-expression network

**Figure 5:**
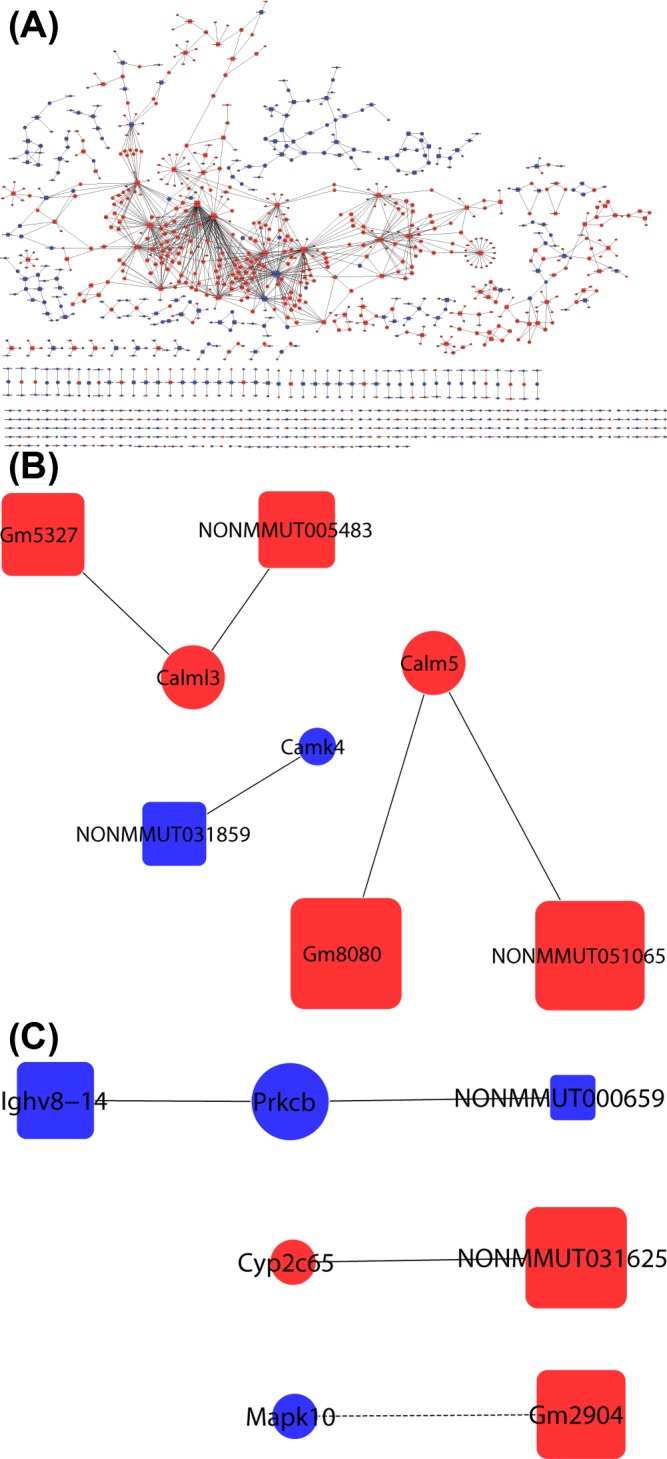
LncRNA–mRNA co-expression network analysis of core mRNAs and their correlated lncRNAs In the network, nodes represent mRNAs, boxes represent lncRNAs, and the size of the node’s area represents the value of betweenness centrality. Red color indicates up-regulation and blue color indicates down-regulation. The lines between nodes indicate a correlative relationship within the group, solid line represents positive correlation, and the dotted line represents negative correlation. (**A**) The whole co-expression network of DE lncRNAs and DE mRNAs. (**B**) Magnified (A) portion; three key mRNAs, Calm5, Calml3, and Camk4 with their associated lncRNAs. (**C**) Magnified (A) portion; the other three key mRNAs with their associated lncRNAs.

The lncRNA–mRNA co-expression network was constructed to identify hub regulatory factors associated with effect of ICA in ApoE^−/−^ mice. The whole profile of co-expression network was made up of 1257 nodes and 1482 connections among 437 DE lncRNAs and 820 DE mRNAs (Figure 5A). According to the key mRNAs selected above in global signal transduction network, we found that lncRNA NONMMUT000659 and Ighv8-14 were positively correlated with Prkcb, lncRNA NONMMUT031625 was positively correlated with Cyp2c65, lncRNA Gm2904 was negatively correlated with Mapk10. LncRNA Gm8080 and NONMMUT051065 were positively correlated with Calm5, lncRNA Gm5327 and NONMMUT005483 were positively correlated with Calml3, lncRNA NONMMUT031859 was positively correlated with Camk4. It was remarkable that the two lncRNAs correlated with Calm5 were among the top five lncRNAs ranked by degree value in the co-expression network (Table 4). Co-expression network analysis of these six key mRNAs and relevant nine lncRNAs are shown in Figures 5B and C.

**Table 4:** The top 5 lncRNAs ranked by degree over 50 after analysis of lncRNA–mRNA co-expression network

Gene symbol	Biotype	Degree	Style
Gm8080	ncRNA	68	Up
NONMMUT069080	ncRNA	67	Down
NONMMUT051065	ncRNA	65	Up
KnowTID_00004804	ncRNA	64	Up
Gm15370	ncRNA	50	Up

### qPCR validation

**Figure 6:**
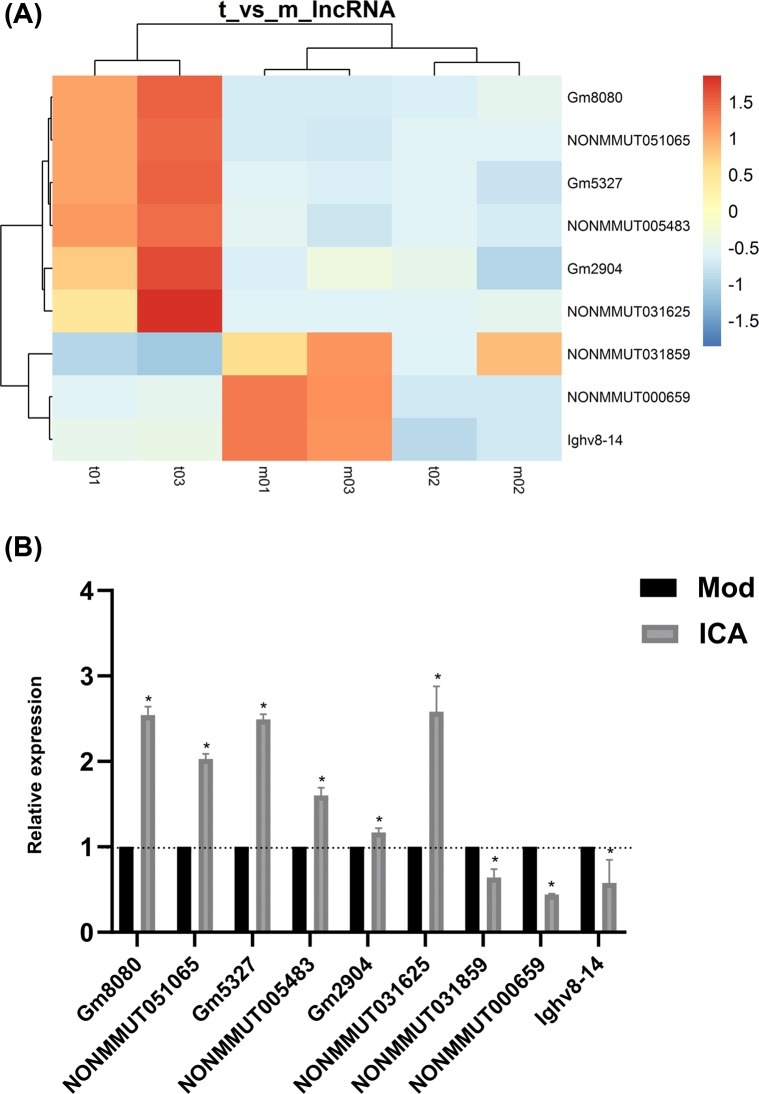
Effect of ICA on the differential lncRNAs expression in ApoE^−/−^ mice (**A**) Heat map from microarray of lncRNAs expression related to key mRNAs. (**B**) qPCR analysis showed that lncRNA Gm8080, NONMMUT051065, Gm5327, NONMMUT005483, Gm2904, and NONMMUT031625 were up-regulated in ICA-treated group compared with model group, whereas NONMMUT031859, NONMMUT000659, and Ighv8-14 were down-regulated (*P<0.05).

**Figure 7:**
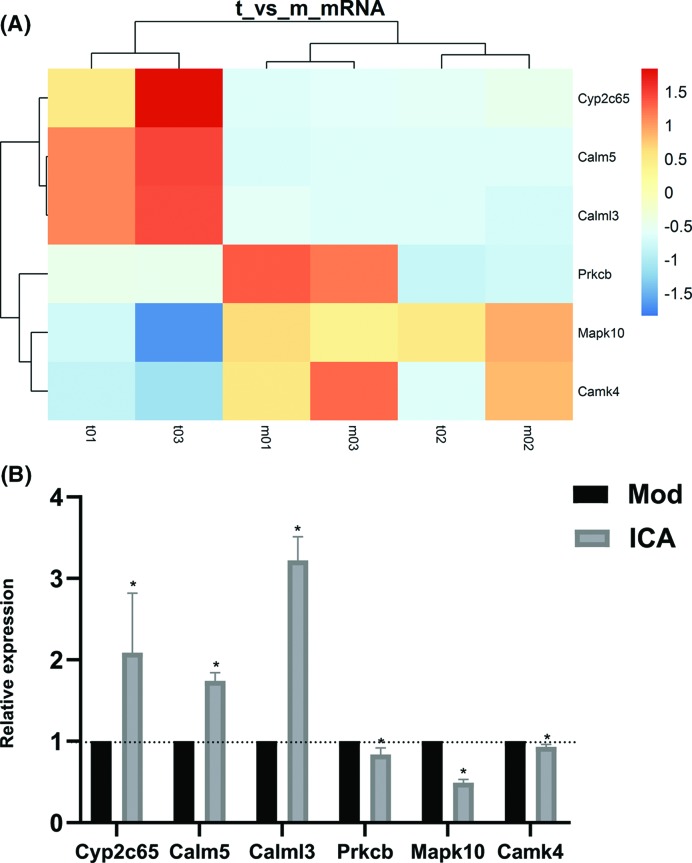
Key mRNAs involved in the action of ICA against AS (**A**) Heat map from microarray of key mRNAs expression. (**B**) qPCR analysis showed that Cyp2c65, Calm5, and Calml3 were up-regulated in ICA-treated group compared with model group, while Prkcb, Mapk10, and Camk4 were down-regulated (*P<0.05).

Six core mRNAs and their correlated nine lncRNAs were chosen for verification of the microarray data by qPCR. The result showed similar trends to those observed in the microarray assay. In detail, the expression of lncRNA Gm8080, NONMMUT051065, Gm5327, NONMMUT005483, Gm2904, and NONMMUT031625 were up-regulated in aorta compared with model group, whereas NONMMUT031859, NONMMUT000659, and Ighv8-14 were down-regulated (Figure 6). Meanwhile, the target mRNAs Cyp2c65, Calm5, and Calml3 were up-regulated, while Prkcb, Mapk10, and Camk4 were down-regulated. Hence, the qPCR data verified the veracity of microarray results (Figure 7).

## Discussion

While we and others have accumulated substantial evidence to suggest that ICA attenuates AS potently, the genetic regulatory mechanism remains to be understood. Moreover, the knowledge of lncRNAs biological function is limited by ICA engaged in anti-AS. In the present study, we clarified that the aortic atherosclerotic lesion area of ApoE^−/−^ mouse model was remarkably reduced by 5.85% with 12-week ICA administration, which was consistent with a previous study [30]. Besides, ICA could also improve lipid metabolism by reducing serum levels of TC and LDL–C.

Nevertheless, no significant differences in serum TG and HDL–C levels were found between groups. The effects of ICA on hyperlipidemia in our study were similar to an earlier publication [31]. Furthermore, we explored the distinct expression profiles of lncRNAs as well as mRNAs in ICA treatment and model ApoE^−/−^ mice using microarray technology, suggesting a large amount of lncRNAs and mRNAs might be linked to the protective effect of ICA. To provide some insights into the biological functions of lncRNAs in anti-atherogenic effect of ICA, we undertook an integrated analysis of lncRNA and mRNA profiling data by bioinformatics approaches. Among the RNA profile, lncRNA NONMMUT031625, Gm2904, Gm8080, NONMMUT051065, Gm5327, NONMMUT005483, NONMMUT000659, Ighv8-14, NONMMUT031859 and their correlated mRNAs Prkcb, Cyp2c65, Mapk10, Calm5, Calml3, Camk4 were selected. Further qRCR was utilized to validate the expression of candidate genes.

GO enrichment and pathway analysis was used to predict the potential function of the DE mRNAs and the corresponding lncRNAs. Functional annotation of mRNAs revealed, for example, DE mRNAs involved in wound healing, positive regulation of osteoblast differentiation, and bone morphogenesis. These critical biological processes were in line with the definite effect of ICA on osteoporosis prevention and bone fractures treatment as one of the primary active ingredients of Herba Epimedii used for centuries [32,33]. Importantly, DE mRNAs were enriched in classical biological processes and pathways which could involve in the pathogenic processes of AS.

The MAPK signal transduction pathways were the most frequently involved contributing to the effects of ICA [34,35]. There are at least three distinct subgroups of MAPKs that are expressed in mammalian cells, which include ERKs (with ERK1 and ERK2 nearly indistinguishable in function), c-Jun NH_2_-terminal kinases (JNKs), and p38 MAPKs [36]. The abnormal proliferation and apoptosis of vascular cell is the pathological basis of AS-related diseases, and the balance between cell proliferation and death is necessary in maintaining homeostasis and normal development of the cells [37]. It is well documented that ERK signaling cascade is capable of modulating cell survival, migration, and apoptosis. Meanwhile, a growing number of studies have reported that ERKs appear to be involved in protecting vascular endothelial cells from apoptosis [38,39]. JNK signaling has been shown to be an integral part of survival pathways in endothelial cells. Investigator pointed out that ICA showed cardiac protective action against cell apoptosis, and the beneficial effect of ICA was JNK-dependent [40,41]. Consistently, our current finding showed that ICA exhibited protective action by regulating important cellular processes such as regulation of ERK1 and ERK2 cascade, and apoptosis. To further explore the core genes among these DE mRNAs in ICA-treated group and controls, we constructed the global signal transduction network, which indicated that plenty of hub genes might play a key role in anti-atherosclerotic effect of ICA. Consequently, as shown in Figure 4 and Table 3, the up-regulated Mapk3 (ERK1) had the second highest degree value and was among the top ten genes ranked by betweenness centrality (degree = 29; betweenness centrality = 0.02755816) in the global signal transduction network. In addition to Mapk3, Mapk10 (JNK3) was among the top 15 genes ranked by degree value (degree = 14; betweenness centrality = 0.0003496) also. These results indicated that ICA might execute anti-apoptotic effect at least partially through ERK and JNK signaling pathway under atherosclerotic pathological conditions.

Modularization contributes to simplifying the intricate network into modules, which are like ‘big genes’. Among those identified, Prkcb was within high range of both degree and betweenness centrality, reflecting its main part in the network. Considerable studies suggested that the activation of protein kinase C (PKC), specifically the β isoform was a critical regulator of AS [42–45]. Yan et al.’s [42] laboratory previously published supportive data that aortas of ApoE^−/−^ mice displayed an age-dependent increase in PKC-βII antigen in membranous fractions, compared with C57BL/6J mice. It was subsequently demonstrated that depletion of PKC-β gene or treatment with PKC-β inhibitor in ApoE^−/−^ mice decreased the atherosclerotic lesion area. Moreover, the activation of JNK was PKCβ-dependent in aortic endothelial cells, which provided mechanistic support of signaling-mediated pathogenesis of AS [42]. On this ground, the same group reported that inhibition of PKC-β reduced CD11c, chemokine (C–C motif) ligand 2 (CCL2), and IL-1β in macrophages induced by high glucose levels.

Their ample evidence uncovered a novel role for PKC-β in modulating CD11c expression and inflammatory response of macrophages in the development of diabetic AS [43].

More recently, Durpe et al.’s [44] study confirmed that PKC-β activation promoted EC dysfunction and monocyte adhesion to the arterial wall, and accelerated atherosclerotic plaque formation in diabetes. Consistently, the aberrantly expressed Prkcb in our result was selected as core mRNA with important function in this study.

LncRNAs are not simply nonfunctional elements but instead are key regulators of gene expression. To identify hub lncRNAs associated with the action of ICA against AS, we constructed lncRNA–mRNA co-expression network. The results showed that the DE lncRNAs interacted with the DE genes, which indicates the complexity of molecular mechanisms for the protective effects of ICA. It was subsequently indicated that the interactions of lncRNA NONMMUT000659—Prkcb and lncRNA Ighv8-14–Prkcb might play crucial roles in ICA-mediated protection against AS.

Subnetworks facilitate the complex network based on microarray method that can be used to identify candidate therapeutic targets. The prominent sub-coexpression-network included five lncRNAs, lncRNA Gm8080, NONMMUT051065, Gm5327, NONMMUT005483, NONMMUT031859, and three mRNAs Calm5, Calml3, Camk4 (Figure 5B). Additionally, two of the five lncRNAs were among the top five lncRNAs ranked by degree value in co-expression network. LncRNAs play important roles in regulation of gene expression, but most lncRNAs are poorly annotated. The interactions of lncRNA–mRNA are helpful in clarifying the biological functions of DE lncRNAs. In global signal transduction network, Camk2d, Calm4, Calm5, Calml3, Calml4, and Camk4 were identified as key genes by degree ranking (Figure 4B). Calcium (Ca^2+^) is known as a secondary messenger in many cellular processes: proliferation, differentiation, and apoptosis. Dysregulation of Ca^2+^ signaling pathway is associated with various disorders, including cerebrovascular diseases, cardiac dysfunction, obesity, aging, and Alzheimer’s diseases. The best studied of the Ca^2+^ signaling proteins is calmodulin (CaM). The structure of CaM-like protein is similar to that of CaM, with four Ca^2+^-binding motifs. Moreover, it has been reported that a number of CaM-like proteins are involved in cell proliferation and apoptosis. Calmodulin-like 3 (CALML3) is useful for normal hyperplastic epidermal development, with a down-regulation of CALML3 expression in potentially malignant phenotype [46]. Overexpression of CALML3 decreased UVB–induced apoptosis and ROS production in cultured human lens epithelial cells (HLECs).

The phosphorylation levels of ERK1/2 were significantly decreased with silencing of CALML3, indicating that ERK1/2 was involved in apoptosis induced by CALML3 down-regulation [47]. Likewise, our bioinformatics analysis showed that some up-regulated DE mRNAs were enriched in regulation of ERK cascade and negative regulation of apoptotic process, some down-regulated DE mRNAs were enriched in the GO term of response to UV (Figure 3A). Many aspects of biological processes are mediated by the formation of a complex with CaM and Ca^2+^-binding proteins (CaBPs). Calm5 is a novel CaBP, which contains four conserved EF-hand motifs (predicted to be Ca^2+^-binding domains) and has homology to CaM-like CaBP genes [48,49]. The functional Ca^2+^-binding domains suggest a probable participation in the regulation of atherogenesis-associated processes through Ca^2+^ signaling-dependent systems. However further experiments are needed to validate this hypothesis.

Calmodulin-dependent kinases (CaMKs) were identified over 20 years ago by activation dependence on Ca^2+^/CaM. CaMKs regulate biological processes that are relevant to cardiovascular disease, by affecting the biology of cardiomyocytes, vascular wall cells and infiltrating macrophages [50,51]. Camk2d (CaMKIIδ) is a regulator of cell proliferation.

Genetic deletion of CaMKIIδ significantly decreased vascular smooth muscle proliferation [52]. Several studies have demonstrated that CaMKII regulates myocardial apoptosis.

CaMKII inhibition protected against apoptosis of cardiomyocytes both *in vitro* and *in vivo* [53]. CaMKIIs have been taken into account while looking at mechanisms of AS [54]. Ozcan et al.’s group previously demonstrated that activation of CaMKIIγ in macrophages could induce apoptosis [55]. Their recent study showed that the increased activation of CaMKIIγ in lesional macrophages was associated with atherosclerotic disease in both humans and mice, and the deletion of myeloid CaMKIIγ in atheroprone mice suppressed development of these advanced plaque characteristics [56]. The expression of Camk4 (CaMKIV) has been demonstrated predominately in cells of immune systems. Several studies suggested that CaMKIV has a key role in the development of Th17 cells and in the modulation of Th17 cells to produce IL-17 [57]. As well, Ichinose et al.’s [58]group implicated that CaMKIV inhibition/silence leads to the suppression of IFN-γ production in T and B cells. AS has been considered as an inflammatory disease of the arterial vessel for years. T and B cells are proposed to be key modulators of atherogenesis and plaque stability. Additionally, IFN-γ is an important regulator of immune function, which is highly expressed in atherosclerotic lesions [59]. These previous investigations were consistent with the functional annotation of key DE mRNAs in our results (Figure 3). Thus, it could be predicted that ICA might be capable of affecting pathogenic processes of AS by suppressing CaMKIV expression. ICA has been reported to have beneficial effects in various conditions. With microarray and bioinformatics methods, the identified CaM-like proteins and CaMKs may be the hub genes affected by ICA when providing its protective activities on AS. Deciphering the related lncRNAs function in the subnetwork may explore new potential targets for AS management.

Taking all these together, we have identified a host of DE lncRNAs and mRNAs in biological activities, which may be pivotal targets of ICA relevant to AS. ICA could mediate multiple cascades against AS, suggesting the pleiotropic effects of ICA in AS alleviation. Our findings would provide a novel insight into gene regulatory network in AS, then further enrich the foundation of knowledge in the extensive pharmacological effects of ICA.

Nonetheless, we recognize that there are several limitations existing in the research which cannot be ignored. First, the effect size of the present research was determined by literature [60,61]; as a consequence, no statistical power analysis was conducted. The sample size was still small in the study, and a larger sample cohort is needed for verification. Second, we have identified the DE genes and associated lncRNAs triggered by ICA, as well as the related cellular process and pathways, nevertheless, the precise molecular mechanisms of these potential regulators in anti-atherogenic functions of ICA are still required to be investigated *in vivo* or *in vitro*. Further studies are still required to explore how ICA modulates the aberrant coding and noncoding RNAs, or which signal molecules lies in the upstream or downstream of the significant pathways. Third, using biological technology we have focused on only a few ‘big genes’ or subnetworks which were relatively rich in information. In particular, detailed information for the complicated interaction network was still unavailable. Apart from the remarkable lncRNAs and mRNAs, most DE genes have not been studied yet. Our current understanding on potential roles of ICA in gene modulation is still in its infancy. Thus, it is imperative to present more reasonable analyses to investigate the complex behavior of ICA.

## Conclusion

In summary, our study demonstrated that ICA was an effective anti-atherosclerotic agent and the action was a complicated process with many lncRNAs, mRNAs and pathways involved. Key genes, such as Prkcb, Cyp2c65, Mapk10, Calm5, Calml3, and Camk4 may be pivotal targets for ICA to combat AS. LncRNA NONMMUT000659, Ighv8-14, NONMMUT031625, Gm2904, Gm8080, NONMMUT051065, Gm5327, NONMMUT005483, NONMMUT031859 were DE lncRNAs triggered by ICA and might play important roles in the development of AS. The present study highlights the benefits of ICA and provides a new insight into the molecular mechanisms of ICA anti-atherogenic function, understanding the intricacies of this process may bring novel avenues for therapuetic intervention. Further molecular intervention experiments are needed to extend the current findings.

## Abbreviations

Adcy2: adenylate cyclase 2
apoE: apolipoprotein E
AS: atherosclerosis
CaBP: Ca^2+^-binding protein
CAD: coronary artery disease
Calm: calmodulin
Calml: calmodulin-like
CaMK: Calmodulin-dependent kinase
Camk2d: calcium/calmodulin-dependent protein kinase II, δ
DE: differentially expressed
Egfr: epidermal growth factor receptor
ERK: extracellular signal-regulated kinase
FC: fold change
GO: Gene Ontology
HDL-C: high-density lipoprotein cholesterol
HFD: high-fat diet
ICA: Icariin
JNK: c-Jun NH_2_-terminal kinase
KEGG: Kyoto Encyclopedia of Genes and Genomes
LDL-C: low-density lipoprotein cholesterol
lncRNA: long noncoding RNA
MAPK: mitogen-activated protein kinase
miRNA: microRNA
ncRNA: noncoding RNA
PCC: Pearson correlation coefficient
PKC: protein kinase C
Prkacb: protein kinase, cAMP dependent, catalytic, β
Prkcb: protein kinase C, β
qPCR: quantitative real-time PCR
TC: total cholesterol
TG: triglyceride
